# TAIMA (Stop) TB: The Impact of a Multifaceted TB Awareness and Door-to-Door Campaign in Residential Areas of High Risk for TB in Iqaluit, Nunavut

**DOI:** 10.1371/journal.pone.0100975

**Published:** 2014-07-17

**Authors:** Gonzalo G. Alvarez, Deborah D. VanDyk, Shawn D. Aaron, D. William Cameron, Naomi Davies, Natasha Stephen, Ranjeeta Mallick, Franco Momoli, Katherine Moreau, Natan Obed, Maureen Baikie, Geraldine Osborne

**Affiliations:** 1 Ottawa Hospital Research Institute, Clinical Epidemiology Program, Ottawa, Ontario, Canada; 2 University of Ottawa, Faculty of Medicine, Ottawa, Ontario, Canada; 3 The Ottawa Hospital, Department of Medicine, Divisions of Respirology and Infectious Diseases, Ottawa, Ontario, Canada; 4 Children’s Hospital of Eastern Ontario, Children’s Hospital of Eastern Ontario Research Institute, Center for Practice Changing Research, Ottawa, Ontario, Canada; 5 Nunavut Tunngavik Inc., Department of Social and Cultural Development, Iqaluit, Nunavut, Canada; 6 Government of Nunavut, Department of Health, Iqaluit, Nunavut, Canada; University of Alabama at Birmingham, United States of America

## Abstract

**Background:**

The incidence rate of active tuberculosis (TB) disease in the Canadian Territory of Nunavut has shown a rising trend over the past 10 years. In 2010 it was 60 times greater than the national incidence rate. The objective of the Taima (translates to “stop” in Inuktitut) TB study was to implement and evaluate a public health campaign to enhance existing TB prevention efforts in Nunavut.

**Methods:**

A TB awareness campaign followed by a door-to-door screening campaign was carried out in Iqaluit, Nunavut. The aim of the campaign was to raise awareness about TB, and to provide in-home screening and treatment for people living in residential areas at high risk for TB. Screening was based on geographic location rather than on individual risk factors.

**Results:**

During the general awareness campaign an increase in the number of people who requested TB testing at the local public health clinic was observed. However, this increase was not sustained following cessation of the awareness campaign. Targeted TB screening in high risk residential areas in Iqaluit resulted in 224 individuals having TSTs read, and detection of 42 previously unidentified cases of latent TB, (overall yield of 18.8% or number needed to screen = 5.3). These cases of latent TB infection (LTBI) were extra cases that had not been picked up by traditional screening practices (34% relative increase within the community). This resulted in a 33% relative increase in the completion of LTBI treatment within the community. The program directly and indirectly identified 5/17 new cases of active TB disease in Iqaluit during the study period (29.5% of all incident cases).

**Conclusions:**

While contact tracing investigations remain a cornerstone of TB prevention, additional awareness, screening, and treatment programs like Taima TB may contribute to the successful control of TB in Aboriginal communities.

## Background

In the early 1960s the incidence rate (per 100,000) of active tuberculosis (TB) disease in the Canadian territory of Nunavut (previously part of the Northwest Territories) was over 1000 [1]. The incidence rates during this period were some of the highest rates of TB ever recorded in history. In the 1960s, public health teams treated entire northern communities with preventive treatment [1], [2], and cases with active TB disease were sent to sanatoriums in the south of Canada in order to prevent further transmission [3], [4]. Over the following 40 years it is believed that through improved social determinants and TB control programs there was a rapid decline in TB incidence, mortality and transmission [1], [5]. However, the incidence rate of active TB disease in Nunavut has shown a rising trend over the past ten years and reached 304 in 2010 (representing 101 active cases) compared to the Canadian rate of 4·6 during the same year [3], [4]. Inuit represent 85·4% of the total population of the territory [6]. Inuit have a disproportionately high rate of TB across Canada compared to other Canadian born Aboriginal people. The incidence rate among First Nations is 22·2/100,000, Métis is 7·5/100,000 versus 198·6/100,000 among Inuit [7].

Between 2005 and 2010, 44% of all of the active cases of TB disease in Nunavut were identified in Iqaluit, the capital of the Territory of Nunavut [4]. In Iqaluit, all of the screening in the community for latent TB infection (LTBI) and most of the investigations for active TB disease are done by the local public health clinic. Community members are screened through contact tracing (active screening), school, employment and walk-ins (passive screening) at the public health clinic. Tracing the contacts of active TB disease cases is a cornerstone of any TB program. Traditional contact tracing focuses on the systematic evaluation of contacts of a known TB patient to identify secondary active cases and recent LTBI cases with the aim to offer treatment to these individuals [3], [8]. Several limitations to traditional contact tracing have been documented in Aboriginal [9] and non Aboriginal communities [10], [11]. In order to overcome some of these limitations, including transmission that occurs outside the home [12], location-based rather than individual-based screening has been done in non Aboriginal communities [13], [14]. Historically, entire Northern Aboriginal communities were screened for TB [4], however this is presently not a feasible approach due to low screening yield and lack of human resources.

The Taima (translates to ‘stop’ in Inuktitut, a dialect of the Inuit language) TB study aimed to implement and evaluate a public health campaign to enhance, but not replace, existing TB prevention programs in Iqaluit, Nunavut. Targeted TB screening was done in residential areas at high risk for TB and was based on geographic location rather than on individual risk factors. The objectives of the study were to evaluate: (1) the impact of a community-wide TB awareness campaign, (2) the efficiency of screening and treatment completion rates from a door-to-door campaign in high risk residential areas as opposed to the entire community, (3) the overall contribution of this approach to the local TB program, and (4) the use of historical high-incidence residential areas in predicting the location of new active TB cases.

## Methods

In 2011, the population of the Territory of Nunavut in the Canadian Arctic was 31,906 [15]. Inuit represent 84% of the total population of the Territory and 60% of the population of Iqaluit [15]. Iqaluit is the capital of Nunavut. It is the largest community in Nunavut with a population of 6,866 people in 2011 [15]. Iqaluit can only be accessed by plane and ship during the brief summer and by plane only during the winter months.

A TB awareness and prevention campaign was designed and carried out by the investigators in Iqaluit, Nunavut between January 13, 2011 and February 28, 2013. The study had three phases. In phase 1, the community at large was engaged with a general TB awareness campaign to provide community members with knowledge about TB, taking into account aspects of the regional culture, language, and TB history. In phase 2, residential areas at high risk for TB within Iqaluit were identified and a door-to-door campaign offering in-home education and screening for TB was undertaken. In phase 3, home treatment was offered when indicated.

**Phase 1** (January to May, 2011) focused on increasing TB awareness and knowledge by engaging the community at large. Community involvement occurred at all levels including the introduction, design, implementation and delivery of the program. Educational TB messaging (a slogan and five TB facts) (http://taimatb.tunngavik.com/) was developed by representatives from Inuit organizations, community members and local TB health care professionals with consideration of the historical Canadian Inuit TB context. Precise translation of the facts into Inuktitut was undertaken because of the number of people who speak Inuktitut in Iqaluit. The TB facts were then tested in a community focus group. The messaging was then integrated into videos made by community members (http://taimatb.tunngavik.com). The videos were then screened at a community feast celebration event where the study and the team members were introduced to the community. Various media events, including a press conference, radio and TV interviews were added to raise community awareness about TB.

**Phase 2** (June to November 2011) was a door-to-door awareness, screening and testing campaign in residential areas that had shown historically high incidence of TB in Iqaluit. Screening of all households in Iqaluit was not feasible. Using public health records, the primary residence of all incident cases of active TB disease during the previous five years was overlaid on a Google maps satellite image of the city. Defined areas were designated from geographically distinct neighbourhoods, and targets for the screening campaign were chosen among those with the highest incident cases per area density (typically those with >5 cases of active TB in the previous five years). Six residential areas were delineated in this fashion. Commercial buildings were excluded. All forms of residential dwellings were considered including apartment buildings and structures housing single or multiple dwellings. All dwellings in these areas were visited via door-to-door screening by a research team consisting of a TB champion and a TB nurse. Three TB champions and three public health nurses were hired for the study. The team did door-to-door screening during working hours, Monday to Friday. The TB champions were local Inuit community members trained in TB education by nursing staff (http://taimatb.tunngavik.com/files/2012/04/TAIMA-TB-Manual.pdf). The videos and TB messaging were then put on a disc to be used as a vehicle to support the oral Inuit tradition for the sharing of information. The videos produced by community members in phase 1 using the five TB facts generated by the community health care staff and lay people were presented in their language of choice (English or Inuktitut) in each household visit using a portable DVD player. This format allowed messaging to be delivered in a standardized and reproducible manner. Discussion and questions surrounding TB, including the signs and symptoms of active TB, were done in Inuktitut and/or English in the home. After providing written informed consent (parents provided consent for children under 16), persons over the age of 6 months were offered LTBI screening in their home using a tuberculin skin test (TST) and an Interferon Gamma Release Assay (IGRA). TSTs were read 48–72 hours later and were considered positive if ≥10 mm of induration. An IGRA was considered positive if >0·35 IU/mL. If either test was positive, then patients were seen in the local clinic and underwent a physician-directed history, physical exam, chest radiograph, and sputum analysis to rule out active TB. Once active TB was ruled out, these patients were offered treatment for latent TB infection.

**Phase 3** (December 2011 to February 2013) participants were offered LTBI treatment. Isoniazid (INH) was administered twice weekly via directly observed preventative treatment (DOPT) for nine months to treat latent TB [16]. Patients were offered home delivery of medications but could choose another location (ex. workplace, school, clinic) if it was more convenient. Treatment assessment was done by local TB doctors. In cases where active TB disease was discovered, participants were transferred to the Iqaluit Public Health TB program for treatment.

### Data analysis

The impact of the phase 1 awareness campaign on the community at large was determined by comparing the number of people who presented passively to the public health clinic before, during and after the general awareness campaign. To determine the efficiency of the phase 2 TB screening in high risk residential areas we compared the yield of new TST positives in the Taima TB program (new TST positive/TST positive+TST negative) to the two previous years of both active and passive screening by the local program (including testing done parallel to the study). To determine the added contribution of Taima TB (phase 2 & 3) to the local public health program, the following was calculated: (1) the number of new TST positives as a proportion of the total number of new TSTs identified during the same time period in the community and (2) the number of persons that started and completed treatment for LTBI as a proportion of the total number of persons that underwent LTBI treatment during the study period in the community.

Ethnicity was determined by land claim beneficiary status. All proportions were compared using the χ^2^ test statistic and Fisher’s exact test, statistical significance at p<0·05. An adjusted logistic regression model was constructed using LTBI as the dependent variable adjusting for covariates: age, sex, smoking, alcohol use, cannabis use and diabetes. All analyses were performed with SAS version 9·2 (SAS, Cary, NC, USA). The study was approved by the Ottawa Hospital Research Institute Ethics Committee and the Public Health Agency of Canada Ethics Board. All patients provided written, informed consent.

## Results

### Impact of awareness campaign on passive LTBI screening in the community

**Figure 1:**
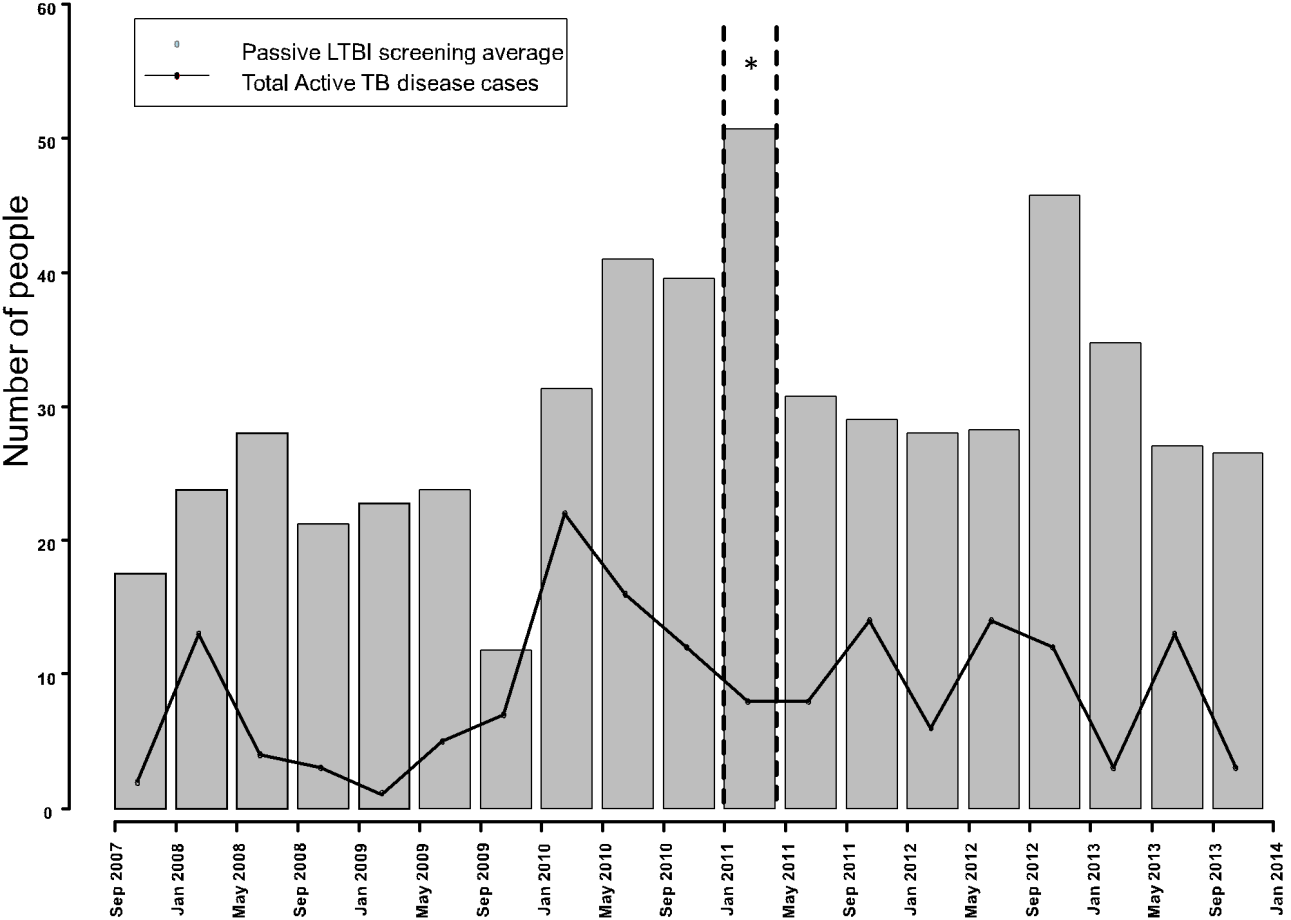
The average number of passive LTBI screenings in 4 month periods prior (10 consecutive periods from 2007–2011) during* (1 period from January to April 2011 inclusive) and after the Taima TB general awareness campaign (8 periods from May 2012 to January 2014 inclusive).

An increase in passive LTBI screening at the local Iqaluit public health clinic was observed during the Taima TB media engagement and general awareness campaign. The number of people who came to the clinic (“walk-ins”) increased from an average of 26 per month over the previous four years to an average of 50 people per month during the four months of Taima TB’s general awareness campaign (p<0·0002). In the four months that followed the general awareness campaign, the number of people who accessed the clinic returned back to an average 24 per month (Figure 1). In the fall of 2012, there was an increase in passive screening that was directly attributable to an increase in TB awareness in the community related to a period of increased disease activity in the schools.

### Population screened

**Figure 2:**
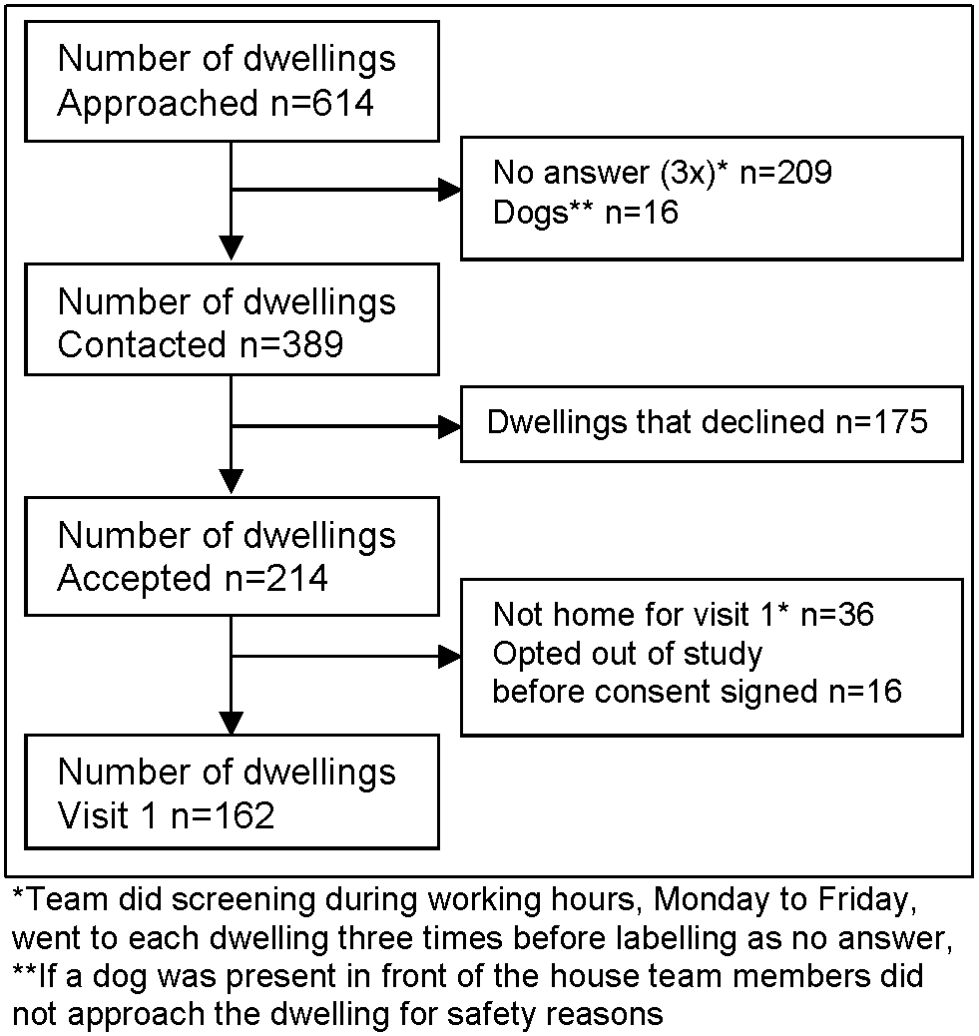
Number of dwellings visited by the Taima TB team during study period (June to November 2011).

**Figure 3:**
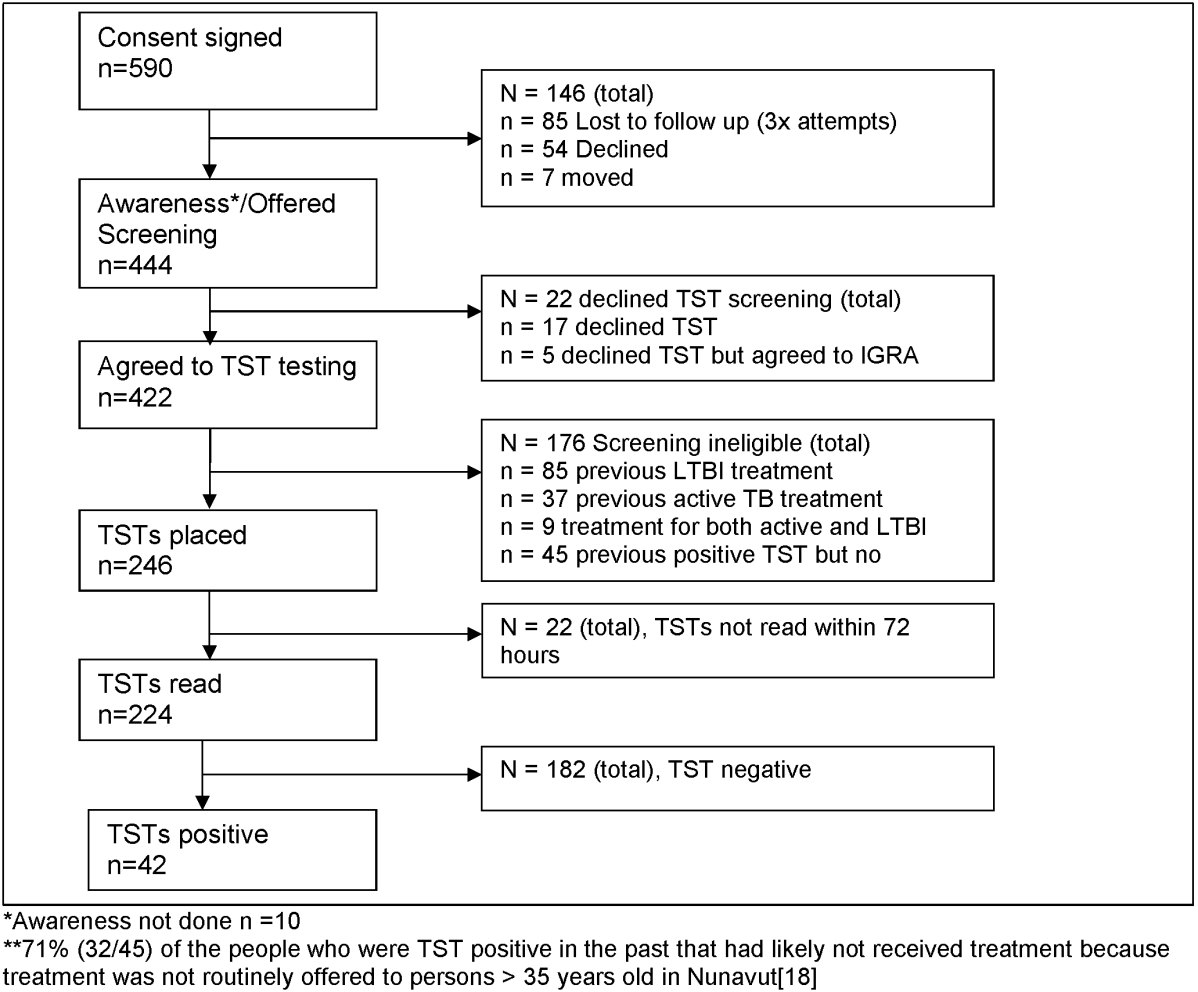
Taima TB participants.

From June to November 2011 the Taima TB program approached 614 dwellings within the identified targeted high-risk screening areas of Iqaluit (Figure 2) which represents 21% of the dwellings in Iqaluit [17], [18]. In 389 of these dwellings, a resident answered the door. Of these 162 dwellings (42%) agreed to participate in the study. Five hundred and ninety individuals signed consent (Table 1). Four hundred and forty-four individuals within these 162 dwellings participated, representing approximately 6% of the population of Iqaluit [15], [17]. Two hundred and forty-six participants had TSTs planted (Table 2), the rest were ineligible for the TST for a variety of reasons (Figure 3). Fifty-nine percent (335/571) of those that consented to enter the study had either never had a TST planted or had not been skin tested for TB within the past 10 years.

**Table 1:** Demographics of Taima TB participants who consented to enter study (n = 590).

Characteristics		N (%)
Age (years)	30 (average)	(1–85) (range)
Sex	Female	329 (56%)
BCG immunization	Yes	396 (67%)
	No	75 (13%)
	Unknown	119 (20%)
TST (at any time)	Yes	460 (78%)
	No	115 (20%)
	Unknown	15 (2%)
TST result	Positive	233 (51%)
	Negative	222 (48%)
	Unknown	5 (1%)
Time since last TST	<10 years	236 (52%)
	≥10 years	220 (48%)
	Unknown	4 (1%)
Previous treatment for LTBI	Yes	146 (25%)
	No	434 (74%)
	Unknown	10 (2%)
Previous treatment for active TB	Yes	65 (11%)
	No	518 (88%)
	Unknown	7 (1%)

**Table 2:** Demographics and pre existing medical risk factors associated with those that were screened for LTBI in the Taima TB program (n = 296*).

Characteristics		N (%)
Age (years)	25 (mean)	(1–82) range
Sex	Female	159 (54%)
Ethnicity	Inuit	251 (86%)
	Canadian born non Aboriginal	45 (14%)
Previous TST**	Never TST	88 (35·7%)
	>10 years	45 (18·2%)
**Risk factors**	**Yes**	**Unanswered**
Present Smoker	119 (40·2%) (64·3%†)	2 (0·7%)
Smoking history	11.5 years (mean)11.2 mean pack years	
Second hand smoke exposure	29 (9·8%)	13 (4·4%)
Alcohol	114 (38·5%) (61·6%†)	9 (3·0%)
Cannabis	86 (29·1%) (46·5%†)	6 (2·0%)
Steroids	0 (0%)	4 (1·4%)
Diabetes	5 (1·7%)	3 (1·0%)
Liver disease	1 (0·3%)	3 (1·0%)
Cancer	1 (0·3%)	3 (1·0%)
HIV	0 (0·0%)	5 (1·7%)

*Includes all persons that had either a TST or IGRA test (n = 246 TSTs placed, n = 45 previous positive TST but no treatment, IGRA done and n = 5 declined TST, only IGRA done).

**Not including those that were already TST positive but had not received treatment.

† Adjusted for persons ≥15 years.

Smoking [OR 7·1, 2·8–18·0 (95% CI)] and age [OR 1·06, 1·03–1·09 (95% CI)] were significant risk factors for LTBI in subjects ≥15 years old after adjusting for sex, current alcohol use, current cannabis use and diabetes (Table 3).

**Table 3:** Multiple logistic regression of persons ≥15 years of age with a positive TST (≥10 mm) who had not received treatment in the past (n = 185).

Characteristic	Odds Ratio (95% CI)	P value
Age (continuous variable)	1·06 (1.03–1.09)	0·0001
Sex (female vs male)	0·69 (0·35–1·39)	0·3
Current smoker†	7·1 (2·8–18·0)	0·0001
Current use of alcohol	1·32 (0.61–2.86)	0·49
Current use of cannabis	1·41 (0.65–3.06)	0·39
Diabetes	0·23 (0.02–2.65)	0·24

† versus non current smoker.

### Impact of door-to-door campaign on screening yield

The Taima door-to-door campaign yielded 42 new cases of previously undiagnosed latent TB. Among those that had the TST planted and read 48–72 hours later, the overall yield of persons with a new positive TST was 18·8% (42/224) (Table 4). An additional six participants had a TST ≥5 mm and another six participants had a negative TST but a positive IGRA. The number of people needed to screen (NNS) (NNS = number of TSTs read/number of new TST positives) to find one person with previously undiagnosed LTBI (new TST positive) was 5·3 people within the Taima TB program (location based screening). For comparison, the NNS within the local public health program was 5·2 people (all reasons for screening included). Contact tracing done by the local program resulted in the most efficient screening approach (NNS of 3·7), followed by walk-in screening (NNS = 5·9) and school and employment screening (both with NNS = 10). During the six month period when the Taima program undertook the door-to-door campaign, there were a total of 123 new positive TST diagnoses identified in Iqaluit (all reasons for screening included). The Taima TB door-to-door campaign contributed 34% of the new LTBI diagnoses (42/123) identified during this period.

**Table 4:** Additional yield of new TST positives obtained during screening in the Taima TB program (location based screening) to the local program (includes all forms of screening) over six month period (June to November 2011).

	Taima TB program	Local TB program
	434*	N/A
TSTs placed	246	524
TSTs read	224 (91.1%)	467 (89.1%)
New TST positives	42 (18.8%)	81 (17·3%)

*received home awareness as part of the Taima TB program, N/A not applicable.

### LTBI treatment completion

In the Taima TB program, 61% of those that were new TST positive diagnoses initiated directly observed prophylactic treatment (at least one dose) compared to the local program where 47% of those that were new TST positive diagnoses initiated treatment. Thirty patients from the Taima TB program completed a full course of treatment for LTBI and took 100% of their expected doses of INH within a 12-month interval. Of these, 17 were new TST positive participants, nine previously TST-positive participants whom had not received treatment in the past and four TST-negative but IGRA-positive participants. During the same six month period the local TB program completed 34 new TST positive treatments and 33 during the same time period the year before. Treatment completion (100% of doses within 12 months) was comparable between both programs for new TST positive diagnoses that took at least one dose of INH (68% (17/25) versus 70·8% (34/48), p value = 0·74). Another three Taima TB participants completed ≥80% (20/25) of doses. The Taima TB program contributed 33% (17/51) of all people in Iqaluit with a new positive TST during the study period who subsequently completed treatment during the study period.

### Active TB case detection

The Taima TB program directly identified three participants with active TB disease. The resulting contact investigations by the local TB team identified another five active cases. The NNS to find one active TB case was 99 (296 participants/three active TB cases). During the six month period of the door-to-door campaign, a total of 17 incident cases of active TB were identified in Iqaluit, of which five (29·5%) were identified as a direct result of Taima TB screening or indirectly through contact investigations of those cases by the local program. Three additional active cases (one Taima TB participant and two contacts) were confirmed after the door-to-door campaign was complete. All together eight cases (six culture confirmed and two children treated as clinical cases) of active TB disease were found directly or indirectly related to the study.

### Residential Areas of High Risk for TB

Eighty two percent (14/17) of the incident cases of active TB that were identified during the course of the study lived within the high risk residential areas that were identified *a priori* by study investigators.

## Discussion

During the general community-wide awareness campaign, an increase in the number of people who requested TB testing (walk-ins/passive testing) at the local public health clinic was observed however this increase was not sustained following the awareness campaign. Targeted TB screening based on location of residence in high-risk areas rather than individual risk factors in high-risk residential areas in Iqaluit resulted in a yield of 18·8% for the detection of new LTBI cases or a NNS of 5·3. The cases of LTBI found were extra cases that would not have been picked up by traditional screening practices. Therefore, the added value of this program in the detection of new LTBI diagnoses and treatment completions to the overall program was an additional 34% and 33% respectively. Furthermore, eight cases of active TB disease were detected in relation to the door-to-door campaign or 29.5% (5/17) of incident cases during the study. The geographical high risk areas identified *apriori* correctly predicted 82% of all cases of active TB disease that occurred within the six month study period.

Individuals who came to the public health clinic as “walk ins” during the community-wide awareness campaign were not asked what triggered their decision to ask for testing. It could be argued that an increase in active TB cases in the community would also result in an increase in people spontaneously walking in for testing even if they had not been identified as contacts. However, during the study period, the number of diagnosed active cases actually decreased. The increase in walk-ins was short lived since once the general awareness campaign was completed the number of walk-ins returned back to the average before the campaign. This finding underscores the need for sustained yearly TB awareness campaigns in the region.

The participants who were screened for TB in residential areas of high risk for TB in Iqaluit were primarily young Inuit, many of whom had never been tested or had not been tested in the previous ten years. This suggests the Taima TB program was able to reach out to people within this high risk group who were not being screened within the traditional local screening programs. When adjusted for age over 15 years, 64% were current smokers with an 11·5 mean pack year history. These tobacco smoking rates are elevated when compared to the rest of Canada (≥15 years, 17% smoke daily) but reflect the Nunavut smoking rates among Inuit (≥15 years, 64% smoke daily [6]). The effect of smoking on risk of LTBI after adjusting for other risk factors remained strong with an elevated OR of 7·1 (95% CI, 2·8–18). Smoking tobacco increases the risk of acquisition of TB infection [19], development of TB disease [20], and is associated with increased TB mortality [21]. This finding underscores the importance of smoking cessation in this population. Age is an expected risk factor as the older a person gets the greater the chance of being exposed to an active TB case [22]. The rate of cannabis use in this cohort was high at 46·5%, in comparison to a random sample of people living in Vancouver who were 40 years or older, where 14% had used cannabis in the previous 12 months [23]. The use of cannabis has also been linked to TB outbreaks [24]. However, in this study, present use of cannabis did not remain significant in multivariate analysis perhaps because duration and quantity of the exposure were not measured. Few participants had pre-existing co-morbidities such as diabetes, liver disease or cancer, likely because of the young age of participants [25]. No cases of HIV were in this cohort.

The high LTBI screening efficiency of the Taima TB program (NNS = 5·3) was comparable to other large studies done in high risk populations (NNS = 5·3 [13], NNS = 5 [14] and NNS = 4 [26]). Furthermore, the NNS to find one active TB case was 99 or 1% (not including those found indirectly as secondary active cases through the local program) which is considerable compared to a recent meta analysis that showed that the pooled prevalence of culture-positive active TB cases among contacts in high-income countries was 0·4% [8]. This finding is concerning because the yield obtained in this study occurred in the presence of continued intense efforts by the local program to do standardized contact tracing around cases of active TB disease in preceding years and even while the study was taking place. The cases identified by the Taima TB program were in addition to those identified by the regular program using standard contact investigations. In a similar study [13] of a door-to-door campaign done in two Texas neighbourhoods at high risk for TB, no cases of active TB were identified during the campaign. However the number of active cases went from 15 cases (1986–1996) before the campaign to zero cases (1996–2006) in the ten years following the campaign. Taken together, these findings suggest that programs like Taima TB have the potential to substantially decrease TB rates in high-risk residential areas. Due to the number of cases in Iqaluit, it may take several rounds of targeted screening in order to achieve important gains in the fight against TB.

The rate of treatment completion is key to any TB public health campaign because the accuracy and yield of the screening test are made obsolete if the participants are unable to complete treatment. Another study [13], had a completion rate of 46% (68/147) for at least six months of self-administered INH; however the definition of initiation of treatment was not defined. In a study that evaluated TB screening in the state of Tennessee [26], 57% of those tested over a four year period initiated self-administered LTBI treatment (received at least one month’s supply of medication). Of those, 54% completed six to nine months of treatment within 12 months. In the Taima TB program, 61% of those that were new TST positive diagnoses initiated directly observed prophylactic treatment (at least one dose). Of those who initiated treatment, 68% completed treatment. In the local program, 47% of those that were new TST positive diagnoses initiated treatment and 70·8% of those that initiated treatment completed treatment. These findings suggest that both the local program and the Taima TB program performed better than other large scale TB screening programs.

### Strengths and limitations

Engaging communities is paramount in TB research [27], [28] and the success of this program is attributable to strong community engagement in the design, development and delivery of the program. This study used fewer technical resources (no geographic information systems (GIS) and no genotyping) making it more applicable and accessible than other studies for front line staff to deploy in remote areas that are underserviced [14]. In Nunavut the extreme temperatures and prolonged darkness in the winter can affect door-to-door campaigns. The Taima TB program required significant resources to execute, given the large number of activities included as part of the research project. Programs looking to implement a similar initiative should choose the activities that best fit their community. Although 23% of the dwellings in Iqaluit were approached to participate in the study, only 25% of those approached participated. However, given the fact that the team went door-to-door during working hours, many people may not have been home (209/614) because they were at work. Of the people who the team was able to contact, 42% accepted entry into the study which is comparable to another study [13] where 50% accepted entry.

## Conclusions

The Taima TB program strengthened existing efforts to reduce TB in Iqaluit. While contact tracing investigations remain a cornerstone of TB prevention, additional awareness, screening, and treatment programs like Taima TB may contribute to the successful control of TB in Aboriginal communities.

## Supporting Information

Definitions S1 (DOCX)
