# Supplementary material for: TAIMA (Stop) TB: The Impact of a Multifaceted TB Awareness and Door-to-Door Campaign in Residential Areas of High Risk for TB in Iqaluit, Nunavut
Source: PLoS One. 2014 Jul 17;9(7):e100975. doi: 10.1371/journal.pone.0100975 (PMC4102461; doi:10.1371/journal.pone.0100975)
Supplement: Definitions S1 — (DOCX) [file pone.0100975.s001.docx]

**Definitions**

Active case finding: when health care providers go door to door (outside the health care services) providing awareness and offer screening for TB (LTBI and active TB) to individuals who would not have been tested under normal circumstances, commonly a health care provider initiated pathway[5].

Active case of TB or TB disease: cases with Mycobacterium tuberculosis complex demonstrated by microscopy and/or on culture or in the absence of bacteriological proof, cases clinically compatible with active TB.

Completion rate: the number of participants completing treatment divided by the number of participants initiating treatment

Directly observed treatment: the supervised ingestion of patients’ prescribed doses, also can be directly observed preventative treatment (DOPT)

Employment screening: TB testing is a condition of employment with some employers. These employees can come to the public health clinic to be tested.

Passive case finding: when an individual comes on their own accord to clinic to be tested for TB (LTBI and active TB), commonly a patient initiated pathway. For LTBI, testing can involve a tuberculin skin test (TST) or a chest radiograph and/or sputums (previously positive TSTs).

School screening: children entering school in Nunavut aged 4 are screened with a TST, all newborns are offered BCG in Nunavut

Started treatment: patient took at least one dose of INH

Residential areas of high risk for TB: areas that had more than 5 active TB cases in the last 5 years
